# A three-step approach for assessing landscape connectivity via simulated dispersal: African wild dog case study

**DOI:** 10.1007/s10980-023-01602-4

**Published:** 2023-02-17

**Authors:** David D. Hofmann, Gabriele Cozzi, John W. McNutt, Arpat Ozgul, Dominik M. Behr

**Affiliations:** 1 Department of Evolutionary Biology and Environmental Studies, University of Zurich, Winterthurerstrasse 190, 8057 Zurich, Switzerland; 2 Botswana Predator Conservation Program, Wild Entrust, Private Bag 13, Maun, Botswana

**Keywords:** Dispersal, Simulation, Movement, Integrated step-selection function, Kavango-Zambezi Transfrontier Conservation Area, Landscape connectivity, Lycaon pictus

## Abstract

**Context:**

Dispersal of individuals contributes to long-term population persistence, yet requires a sufficient degree of landscape connectivity. To date, connectivity has mainly been investigated using least-cost analysis and circuit theory, two methods that make assumptions that are hardly applicable to dispersal. While these assumptions can be relaxed by explicitly simulating dispersal trajectories across the landscape, a unified approach for such simulations is lacking.

**Objectives:**

Here, we propose and apply a simple three-step approach to simulate dispersal and to assess connectivity using empirical GPS movement data and a set of habitat covariates.

**Methods:**

In step one of the proposed approach, we use integrated step-selection functions to fit a mechanistic movement model describing habitat and movement preferences of dispersing individuals. In step two, we apply the parameterized model to simulate dispersal across the study area. In step three, we derive three complementary connectivity maps; a heatmap highlighting frequently traversed areas, a betweenness map pinpointing dispersal corridors, and a map of inter-patch connectivity indicating the presence and intensity of functional links between habitat patches. We demonstrate the applicability of the proposed three-step approach in a case study in which we use GPS data collected on dispersing African wild dogs (*Lycaon pictus*) inhabiting northern Botswana.

**Results:**

Using step-selection functions we successfully parametrized a detailed dispersal model that described dispersing individuals’ habitat and movement preferences, as well as potential interactions among the two. The model substantially outperformed a model that omitted such interactions and enabled us to simulate 80,000 dispersal trajectories across the study area.

**Conclusion:**

By explicitly simulating dispersal trajectories, our approach not only requires fewer unrealistic assumptions about dispersal, but also permits the calculation of multiple connectivity metrics that together provide a comprehensive view of landscape connectivity. In our case study, the three derived connectivity maps revealed several wild dog dispersal hotspots and corridors across the extent of our study area. Each map highlighted a different aspect of landscape connectivity, thus emphasizing their complementary nature. Overall, our case study demonstrates that a simulation-based approach offers a simple yet powerful alternative to traditional connectivity modeling techniques. It is therefore useful for a variety of applications in ecological, evolutionary, and conservation research.

**Graphical abstract:**

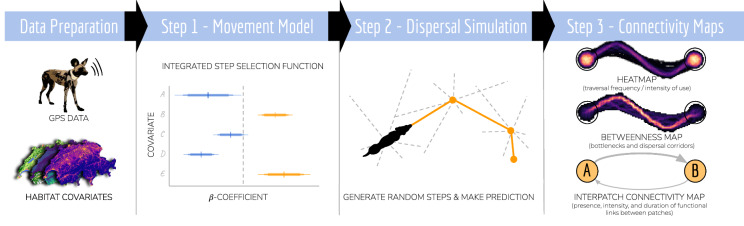

**Supplementary Information:**

The online version contains supplementary material available at 10.1007/s10980-023-01602-4.

## Introduction

Dispersal of individuals is a vital process that allows species to maintain genetic diversity (Perrin and Mazalov 2000; Frankham et al. 2002; Leigh et al. 2012; Baguette et al. 2013; LaPoint et al. 2013), rescue non-viable populations (Brown and Kodric-Brown 1977), and to colonize unoccupied habitats (Hanski 1999; MacArthur and Wilson 2001). However, the ability to disperse depends on a sufficient degree of landscape connectivity (Fahrig 2003; Clobert et al. 2012), making the identification and protection of dispersal corridors that promote connectivity a task of fundamental importance (Doerr et al. 2011; Rudnick et al. 2012). Identifying dispersal corridors not only necessitates a comprehensive understanding of the factors that limit dispersal, but also an appropriate model to estimate connectivity (Baguette et al. 2013; Vasudev et al. 2015; Hofmann et al. 2021a). To date, the most commonly used connectivity models are least-cost path analysis (LCPA; Adriaensen et al. 2003) and circuit theory (CT; McRae 2006; McRae et al. 2008). Unfortunately, both models rest on assumptions that appear unsuitable for dispersers, thus calling for the development of alternative approaches. One promising alternative is to assess landscape connectivity via simulated dispersal trajectories generated from individual-based movement models (IBMMs, Diniz et al. 2019). However, IBMMs require a large number of subjective modeling decisions, thus making among-system comparisons difficult.

Traditional connectivity models make assumptions that are rarely met for dispersers. LCPA, for instance, assumes that individuals move towards a preconceived endpoint and choose a cost-minimizing route accordingly (Sawyer et al. 2011; Abrahms et al. 2017). While this assumption may be justifiable for migrating animals, it is unlikely to hold for dispersers, as dispersers typically move across unfamiliar territory towards an unknown endpoint (Koen et al. 2014; Cozzi et al. 2020). CT, on the contrary, posits that animals move according to a random walk, entailing that autocorrelation between subsequent movements cannot be rendered (Diniz et al. 2019). For dispersers, however, autocorrelated movements are regularly observed (Cozzi et al. 2020; Hofmann et al. 2021a), meaning that dispersal trajectories are usually strongly directional. An interesting generalization that bridges the continuum between LCPA and CT has been proposed by Panzacchi et al. (2016) and enables to capitalize on the merits of both approaches. Despite these and several other generalizations of LCPA and CT (e.g. Pinto and Keitt 2009; Landguth et al. 2012; Panzacchi et al. 2016; Brennan et al. 2020), some shortcomings remain. Most notably, all of these methods rely on static permeability or resistance surfaces that can’t reflect the temporal dimension of dispersal. This permits statements about the expected duration for moving between habitat patches (Martensen et al. 2017; Diniz et al. 2019).

The shortcomings inherent to LCPA and CT can be overcome by simulating dispersal using IBMMs and by converting simulated trajectories into meaningful measures of connectivity (Diniz et al. 2019). In contrast to LCPA and CT, IBMMs allow to explicitly simulate how individuals move across and interact with the encountered landscape (Kanagaraj et al. 2013; Clark et al. 2015; Allen et al. 2016; Hauenstein et al. 2019; Zeller et al. 2020), as well as to render potential interactions between movement behavior and habitat conditions (Avgar et al. 2016). This shifts the focus towards a more functional view on connectivity (Tischendorf and Fahrig 2000). Furthermore, IBMMs generate movement sequentially, i.e. they generate a series of steps, so that the temporal dimension of dispersal becomes explicit and allows modeling autocorrelation between successive steps (Diniz et al. 2019). Finally, simulations from IBMMs do not enforce movement or connections towards preconceived endpoints but allow individuals to adjust their route “on the go”, thereby preventing biases arising from misplaced endpoints. Despite these advantages, a unifying approach to simulate dispersal and assess connectivity using IBMMs is lacking. Considering the large number of subjective decisions entailed by IBMMs, an approach that streamlines and standardizes the application of dispersal simulations to assess connectivity will, however, be critical to safeguard comparability among studies.

**Fig. 1:**
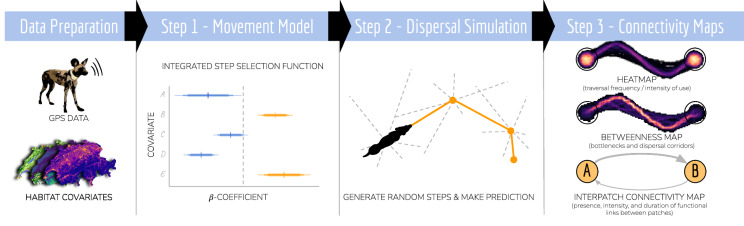
Flowchart of the simulation-based connectivity analysis. First, GPS data and habitat covariates must be collected. The combined data is then analyzed using an integrated step-selection model (step 1). The parametrized model is then treated as an individual-based movement model and used to simulate dispersal trajectories (step 2). Ultimately, simulated trajectories are translated into a set of maps that are pertinent to landscape connectivity (step 3). This includes a heatmap, indicating the traversal frequency across each spatial unit of the study area, a betweenness map, highlighting movement corridors and bottlenecks, and, finally, an inter-patch connectivity map, where the frequency of connections and their average duration can be depicted

Here, we propose and exemplify a simple three-step approach for simulating dispersal and assessing landscape connectivity (Fig. 1). In step one, we combine GPS movement data of dispersing individuals with habitat covariates to fit a mechanistic movement model via integrated step-selection functions (ISSFs, Avgar et al. 2016). We chose to use ISSFs because the framework not only allows inference on the study species’ habitat kernel (i.e. its habitat preferences), but also its movement kernel (i.e. its movement preferences/capabilities) and potential interactions among the two (Avgar et al. 2016; Fieberg et al. 2021). In step two, we use the parametrized movement model to simulate dispersal across the study area. Comparable simulations have already been applied to estimate steady-state utilization distributions of resident individuals (Potts et al. 2013; Signer et al. 2017) and to model landscape connectivity, yet disregarding interdependencies between habitat and movement kernels (Clark et al. 2015; Zeller et al. 2020). Finally, in step three, we convert the simulated trajectories into three complementary connectivity maps; (i) a heatmap revealing frequently traversed areas (e.g. Hauenstein et al. 2019; Zeller et al. 2020), (ii) a betweenness-map delineating dispersal corridors and bottlenecks (e.g. Bastille-Rousseau et al. 2018), (iii) and a map of inter-patch connectivity, depicting the presence and intensity of functional links between habitat patches, as well as the average dispersal duration required to realize those connections (e.g. Gustafson and Gardner 1996; Kanagaraj et al. 2013).

We showcase the application of the proposed approach using GPS movement data collected on dispersing African wild dogs (*Lycaon pictus*). The African wild dog is a highly mobile species whose population persistence heavily relies on the availability of large, natural or semi-natural landscapes and a sufficient degree of connectivity among remaining subpopulations. Once common throughout sub-Saharan Africa, this species has disappeared from much of its historic range, largely due to human persecution, habitat fragmentation, and disease outbreaks (Woodroffe and Sillero-Zubiri 2012). Wild dogs typically disperse in single-sex coalitions (McNutt 1996; Behr et al. 2020) and are capable of dispersing several hundred kilometers (Davies-Mostert et al. 2012; Masenga et al. 2016; Cozzi et al. 2020). Although previous studies have investigated connectivity for this species using LCPA (Hofmann et al. 2021a) and CT (Brennan et al. 2020), a more comprehensive and mechanistic understanding of dispersal and connectivity is missing [but see Creel et al. (2020)]. Nevertheless, with about 6,000 free-ranging wild dogs remaining in fragmented subpopulations (Woodroffe and Sillero-Zubiri 2012), reliable information on dispersal behavior and landscape connectivity is essential for the conservation of this endangered carnivore. We anticipated that a connectivity assessment based upon our three-step approach would overcome several of the conceptual shortcomings of traditional connectivity models, while providing a more detailed view on movement behavior during dispersal its implications for landscape connectivity.

## Methods

### Case study

#### GPS data

We applied the three step approach presented in Fig. 1 to GPS movement data from 16 dispersing African wild dog coalitions (7 female and 9 male coalitions). This data has been collected between 2011 and 2019 from a free-ranging wild dog population in northern Botswana. During dispersal, GPS collars recorded a fix every 4 h and regularly transmitted data over the Iridium satellite system. To ensure comparable time intervals between GPS fixes, we removed any fixes that were not successfully obtained at the desired 4-hour schedule (allowing for a tolerance of $\pm$ 15 min). To prepare the data for step-selection analysis, we converted the fixes (n = 4’169) into steps, where each step represented the straight-line movement between two consecutive GPS fixes (Turchin 1998). We only considered steps with equal step-durations (i.e. 4 h) for further analysis. We will refer to these steps as “realized steps”. We did not differentiate between sexes, for previous research found little differences between sexes during dispersal (Woodroffe et al. 2020; Cozzi et al. 2020). Additional details on the data collection and preparation can be found in Cozzi et al. (2020) and Hofmann et al. (2021a).

#### Study area

**Fig. 2:**
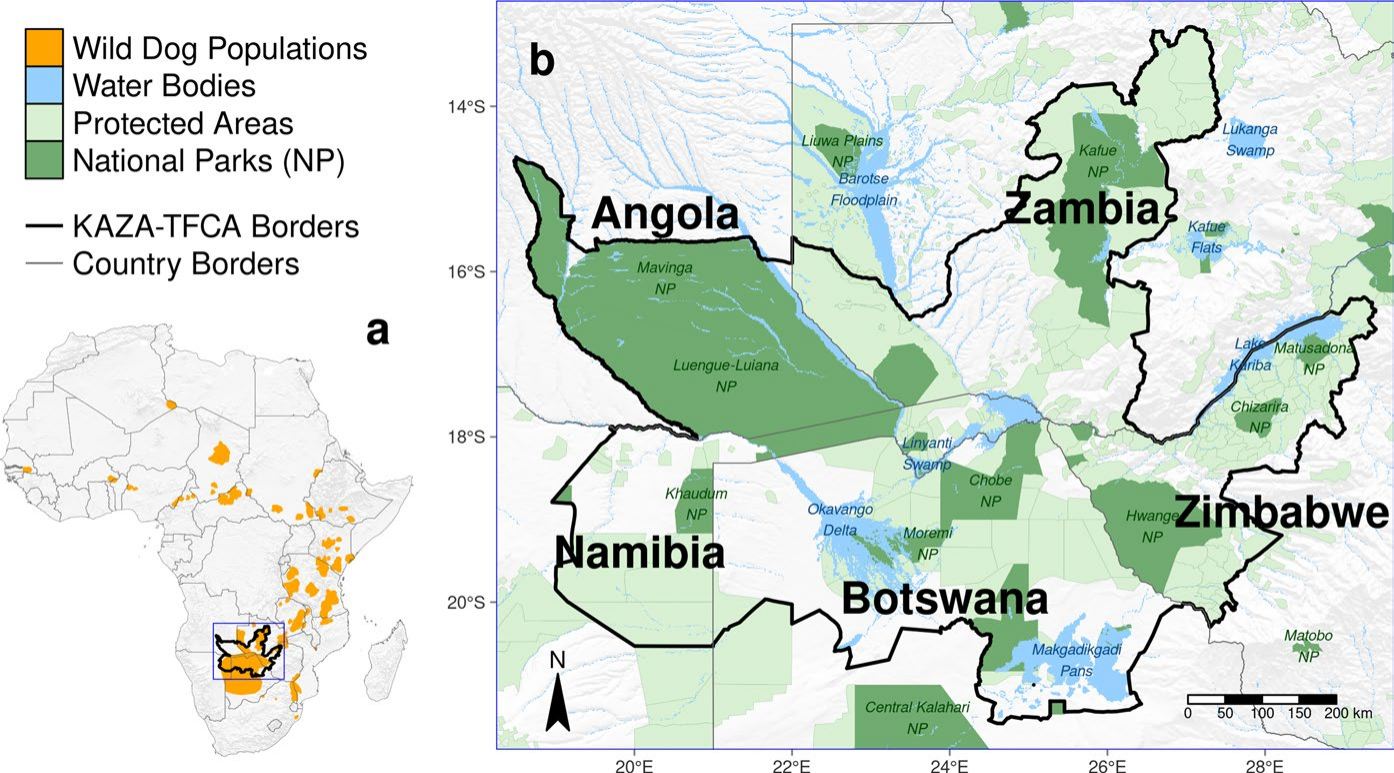
Illustration of the study area in southern Africa. **a** The study area was confined by a bounding box spanning the entire KAZA-TFCA which comprises parts of Angola, Namibia, Botswana, Zimbabwe, and Zambia. Data on remaining wild dog populations (orange) has been sourced from Woodroffe and Sillero-Zubiri (2012). **b** The KAZA-TFCA represents the world’s largest terrestrial transfrontier conservation area and covers a total area of 520’000 km$^2$. Its main purpose is to re-establish connectivity between already-existing NPs (dark green) and other protected areas (light green)

Our simulation of dispersal trajectories and assessment of connectivity spanned across the entire Kavango-Zambezi Transfrontier Conservation Area (KAZA-TFCA, Fig. 2a, b) and encompassed a rectangular extent of roughly 1.3 Mio. km$^2$. With an area of 520’000 km$^2$, the KAZA-TFCA is the world’s largest transboundary conservation area and comprises parts of Angola, Botswana, Namibia, Zimbabwe, and Zambia, thus hosting a rich diversity of landscapes, ranging from savannah to grassland and from dry to moist woodland habitats. In its center lies the Okavango Delta, a dominant hydro-geographical feature and the world’s largest flood-pulsing inland delta. Large portions of the KAZA-TFCA are formally protected in the form of national parks (NPs) or other protected areas, yet a considerable portion of the landscape remains human-dominated (e.g. roads, agricultural sites, and settlements).

#### Habitat covariates

We represented the physical landscape in our study area by the habitat covariates water-cover, distance-to-water, woodland-cover, shrub/grassland-cover, and human-influence. To render the seasonal dynamics of water-cover for the extent of the Okavango Delta, we applied an algorithm that enabled us to obtain weekly updated raster-layers for water-cover and distance-to-water from MODIS satellite imagery (Wolski et al. 2017; Hofmann et al. 2021a). This algorithm is now implemented in the floodmapr package (available on GitHub; https://github.com/DavidDHofmann/floodmapr). To ensure a consistent resolution across habitat covariates, we coarsened or interpolated all layers to a resolution of 250 m x 250 m. A detailed description of how we prepared each habitat covariate is provided in Hofmann et al. (2021a).

We performed all data preparations, spatial computations, and statistical analysis in R, version 4.2.2 (Core Team 2022). Some helper functions were written in C++ and imported into R using the Rcpp package (Eddelbuettel and François 2011; Eddelbuettel 2013; Eddelbuettel and Balamuta 2018).

### Step 1—movement model

We combined the collected GPS data with habitat covariates and used ISSFs (Avgar et al. 2016) to parametrize a mechanistic movement model. More specifically, we paired each realized step with a set of 24 randomly generated alternative steps. A realized and its 24 random steps together formed a stratum that received a unique identifier. As suggested by Avgar et al. (2016), we generated random steps by sampling random turning angles from a uniform distribution ($-\pi , +\pi$) and step lengths from a gamma distribution that was fitted to realized steps (scale $\theta$ = 6’308 and shape $k$ = 0.37). Note that our approach of sampling turning angles from a uniform distribution does not imply that we assume uniform turning angles, as we will account for directionality later in the model (Avgar et al. 2016; Fieberg et al. 2021).

Along each realized and random step, we extracted values from underlying habitat covariate layers and we computed averages of each covariate along the steps. Besides extracting *habitat covariates*, we also computed movement metrics that we used as *movement covariates* in the ISSF models (Avgar et al. 2016; Fieberg et al. 2021). Specifically, we computed the step length (sl), its natural logarithm (log(sl)), and the cosine of the relative turning angle (cos(ta)), which is a measure of directionality (Turchin 1998), for each step. Because wild dog activity is low during the hot midday hours (Cozzi et al. 2012), we additionally created the variable LowActivity, indicating whether a step was realized during periods of low wild dog activity (09:00 to 17:00 local time) or high wild dog activity (17:00 to 09:00 local time). To facilitate model convergence, we standardized all continuous covariates to a mean of zero and a standard deviation of one. Correlations among covariates were low [$|r| < 0.6$; Latham et al. (2011)], so we retained all of them for modeling.

To contrast realized steps (scored 1) and random steps (scored 0), we assumed that animals assigned a selection score $w(x)$ to each step [Eq. 1; Fortin et al. (2005)], where $w(x)$ depended on the step’s associated covariates ($x_1, x_2,..., x_n$) and on the animal’s relative selection strengths (Avgar et al. 2017) towards these covariates ($\beta _1, \beta _2,..., \beta _n$):

1 $$\begin{aligned} w(x) = exp(\beta _1 x_1 + \beta _2 x_2 + \cdots + \beta _n x_n) \end{aligned}$$

The probability of a step $i$ being realized was then contingent on the step’s selection score, as well as on the selection scores of all other step in the same stratum:

2 $$\begin{aligned}{} & {} P(Y_{i} = 1 | Y_{1} + Y_{2} + \cdots + Y_{i} = 1) \nonumber \\{} & {} \quad = \frac{w(x_{i})}{w(x_{1}) + w(x_{2}) + \cdots + w(x_{i})} \end{aligned}$$

To estimate relative selection strenghts (i.e. the $\beta$-coefficients), we used mixed effects conditional logistic regression analysis, implemented through the r-package glmmTMB (Brooks et al. 2017). The implementation of conditional logistic regression has been proposed by Muff et al. (2020) and allows to model random slopes. The method requires to fix the variance of the stratum specific intercept to a large value, so we fixed it to an arbitrary high value of $10 ^ 6$ and used disperser identity to model random slopes for all covariates.

Our movement model was based on a habitat selection model that was previously developed for dispersing wild dogs [hereafter referred to as *base model*, Hofmann et al. (2021a)]. In the base model, no interactions among habitat covariates and movement covariates were considered, so we here expanded the model and allowed for such interactions, acknowledging that movement preferences during dispersal could depend on habitat conditions (details in Online Appendix A1). To determine the most parsimonious movement model among model candidates, we ran stepwise forward model selection based on Akaike’s Information Criterion (AIC, Burnham and Anderson 2002)]. More specifically, we started with the base model and iteratively increased model complexity by adding all possible interactions between movement and habitat covariates. Given that the focus of our analysis lied on predicting dispersal patterns and all model candidates were biologically intuitive, we deemed the use of model selection appropriate. However, caution should be employed if causal relationships are of interest, as model selection may lead to biased parameter estimate (Whittingham et al. 2006). We validated the predictive power of the most parsimonious model using k-fold cross-validation for case–control studies as described in Fortin et al. (2009). This validation attests significant prediction ability to the movement model if the model outperforms a random guess and systematically assigns low ranks (high selection scores) to observed steps (details in Online Appendix A2).

### Step 2—dispersal simulation

We used the most parsimonious movement model to simulate individual dispersal trajectories across the study area. The simulation of a dispersal trajectory resembled an “inverted” ISSF and was set up as follows. (1) We defined a source point and assumed a random initial orientation of the simulated animal. (2) Starting from the source point, we generated 25 random steps by sampling turning angles from a uniform distribution ($-\pi , +\pi$) and step lengths from our fitted gamma distribution. (3) Along each random step, we extracted and averaged values from the habitat covariate layers and we computed the movement metrics sl, log(sl), and cos(ta). To ensure compatible scales with the fitted movement model, we standardized covariate values using means and standard deviations from the empirical data. (4) We applied the parametrized movement model to predict the selection score $w(x)$ for each step using Eq. 1 and we converted predicted scores into probabilities using Eq. 2. (5) We randomly sampled one of the generated random steps based on assigned probabilities and determined the animal’s new position. We repeated steps (2) to (5) until 2’000 steps were realized and we repeated the simulation until a total of 80’000 dispersal trajectories was reached.

As source points for the simulations, we distributed 50’000 points at random locations inside protected areas that were large enough to host an average size wild dog home range [i.e. > 700 km$^2$; Pomilia et al. (2015)]. We placed another 30’000 points randomly inside the buffer zone, mimicking potential immigration into the study area (Fig. S1). To mitigate edge effects and to deal with random steps leaving the study area, we followed Koen et al. (2010) and artificially expanded all covariate layers by a 100 km wide buffer zone. Within the buffer zone, we randomized covariate values by resampling values from the original covariate layers. Through this buffer zone, simulated dispersers were able to leave and re-enter the main study area. In cases where random steps crossed the outer border of this buffer zone, we resampled steps until they fully lied within the buffer zone, essentially forcing simulated individuals to remain within the expanded study area.

To ensure reliable connectivity estimates, we determined the number of simulated dispersal trajectories required to reach a “steady state”. For this purpose, we distributed 1’000 rectangular “checkpoints”, each with an arbitrary extent of 5 km $\times$ 5 km, at random coordinates within the study area (excluding the buffer). We then determined the relative frequency at which each checkpoint was traversed by simulated dispersal trajectories (hereafter referred to as relative traversal frequency) as we gradually increased the number of simulated trajectories from 1 to 50’000. To assess variability in the relative traversal frequency, we repeatedly subsampled 100 times from all 50’000 trajectories and computed the mean traversal frequency across replicates, as well as its 95% prediction-interval for each checkpoint. We considered connectivity to have reached a steady state once the width of the prediction-interval dropped below a value of 0.01 for all checkpoints.

### Step 3—connectivity maps

#### Heatmap

To identify dispersal hotspots within the study area, we created a heatmap indicating the absolute frequency at which different areas were traversed by simulated dispersal trajectories (e.g. Hauenstein et al. 2019; Zeller et al. 2020). Specifically, we rasterized all simulated trajectories onto a raster with 1 km $\times$ 1 km resolution and tallied resulting layers into a single map. This procedure ensured that every trajectory was only counted once, even if it traversed the same raster-cell multiple times, thus reducing potential biases caused by individuals that were surrounded by unfavorable habitat and “moved in circles”. To achieve high performance rasterization, we used the R-package terra (Hijmans 2021). For a subset of the study area, we also generated heatmaps at 250 m $\times$ 250 m, yet found little qualitative differences to the coarser resolution, thus suggesting the choice of 1 km $\times$ 1 km to be appropriate.

#### Betweenness map

To pinpoint movement corridors and bottlenecks, we converted simulated trajectories into a network and calculated betweenness scores for all raster-cells in the study area (Bastille-Rousseau et al. 2018). Betweenness is a pertinent metric for connectivity as it measures how often a specific network-node (in our case a raster-cell) lies on a shortest path between any other pair of nodes (Bastille-Rousseau et al. 2018). To convert simulated trajectories into a network, we followed Bastille-Rousseau et al. (2018) and overlaid the study area (including the buffer) with a raster containing 2.5 km $\times$ 2.5 km raster-cells, where the center of each raster-cell served as node in the final network. To identify edges (i.e. connections) between the nodes, we used the simulated trajectories and determined all transitions occurring from one cell to another, as well as the frequency at which those transitions occurred (see also Online Appendix A4). This resulted in an edge-list that we translated into a weighted network using the r-package igraph (Csardi and Nepusz 2006). The final weight of each edge was determined by the frequency of transitions, yet because igraph handles edge weights ($\omega$) as costs, we inverted the traversal-frequency through each raster-cell by applying $\omega = \frac{mean\,(Traversal \,Frequency)}{Traversal \,Frequency_i}$. Consequently, regularly used edges received small weights (i.e. low costs) and vice versa. We used the weighted network to calculate betweenness scores for all network nodes.

#### Inter-patch connectivity map

To examine the presence and intensity of functional links (i.e. connections) between patches within the study area, we calculated inter-patch connectivity (e.g. Gustafson and Gardner 1996; Kanagaraj et al. 2013). For this, we computed the relative frequency at which dispersers originating from one patch successfully moved into another patch. We considered movements between patches as successful if an individual’s dispersal trajectory originating from the source patch intersected with the target patch at least once. For each trajectory we also recorded the number of steps required to reach the first intersection with the respective patch, allowing us to compute the average dispersal durations from one patch to another. In summary, we determined *if* and *how often* dispersers moved between certain patches, as well as *how long* individuals had to move to make these connections. In our case study, we used NPs as patches to determine inter-patch connectivity, hence we’ll use the terms interchangeably from here on. The decision to focus on NPs was purely out of simplicity and should not imply that dispersal between other areas is impossible.

#### Validation

To validate our predictions of connectivity, we utilized additional dispersal data that was collected on eight dispersing coalitions between 2019 and 2022 (totalling to 2’668 GPS locations). We used a path selection function [PSF, Cushman and Lewis (2010)] to assess if observed dispersal trajectories followed areas of high predicted connectivity. Similar to SSF, PSF enables to detect selection for certain features by comparing observed paths to randomly generated paths. Here, we paired each observed path with 50 random paths that we generated by randomly rotating and shifting observed paths by a random angle $\alpha \sim U(-\pi , +\pi )$ and a random distance $d \sim U(0$ km, $50$ km). Along each path, we then extracted connectivity values from the heatmap (see above) generated after 68, 125, 250, 500, and 2’000 simulated steps, respectively. Finally, we ran conditional logistic regression to contrast observed and random paths. In case of systematic selection for high-connectivity areas, the regression coefficients from the corresponding conditional logistic regression model should be positive.

## Results

**Fig. 3:**
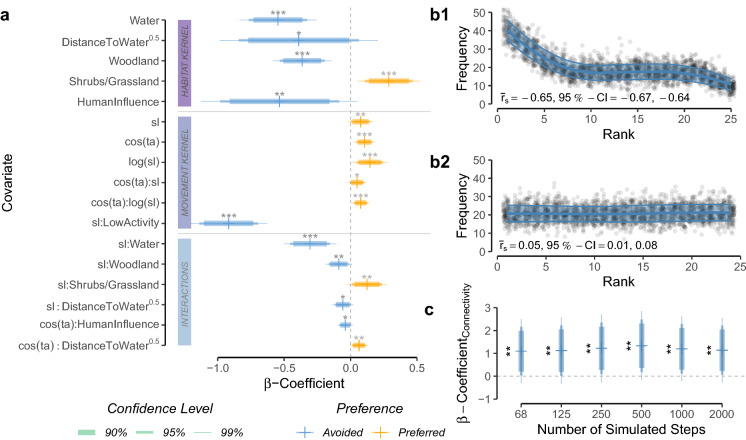
**a** Most parsimonious movement model for dispersing wild dogs. The model comprises a habitat kernel, a movement kernel, as well as their interactions. The horizontal line segments delineate the 90%, 95%, and 99% confidence-intervals for the respective $\beta$-coefficients. Significance codes: * $p < 0.10$, ** $p < 0.05$, *** $p < 0.01$. **b** Results from the k-fold cross-validation procedure. Subfigure **b1** shows rank frequencies of realized steps according to model predictions with known preferences, whereas subfigure **b2** shows rank frequencies of realized steps when assuming random preferences. The blue ribbon shows the prediction interval around a loess smoothing regression that we fitted to ease the interpretation of the plots. The significant correlation between rank and associated frequency in **b1** highlights that the most parsimonious model successfully outperformed a random guess **b2** and frequently assigned low ranks (i.e. high selection scores) to realized steps but only rarely high ranks (i.e. low selection scores). **c** Results from the PSF analysis using independent dispersal data show that dispersers preferrably moved through areas where our heatmaps predicted high connectivity. Results are shown for heatmaps realized after 68, 125, 250, 500, and 2’000 simulated steps, respectively

The most parsimonious movement model consisted of movement covariates, habitat covariates, as well as several of their interactions, thus suggesting that movement behavior during dispersal depended on habitat conditions (Fig. 3a, Tables S1 and S2). Although multiple models received an AIC weight > 0 (Table S1), we only considered results from the most parsimonious model for simplicity. This decision only marginally influenced subsequent steps as all models with positive AIC weights retained similar covariates (Table S1). The k-fold cross-validation showed that the final model substantially outperformed a random guess and provided reliable predictions (i.e. confidence intervals of ${\bar{r}}_{s, realized}$ and ${\bar{r}}_{s, random}$ did not overlap). Moreover, the model correctly assigned high selection scores to realized steps (Fig. 3b), indicating a good fit between predictions and observations. As can be taken from the Spearman rank correlation coefficient, the inclusion of several interactions between movement and habitat covariates significantly improved model performance $({\bar{r}}_{s, realized} = -0.65; 95\%-CI = [-0.67, -0.64])$), compared to the base model [${\bar{r}}_{s, realized} = -0.55; 95\%-CI = [-0.57, -0.52]$; Hofmann et al. (2021a)]. Our validation of the resulting connectivity maps using independent dispersal data showed that dispersers preferentially followed areas of high predicted connectivity, as coefficients from the PSF models were all significantly greater than zero (Fig. 3c). The movement model thus successfully predicted functional connectivity.

Plots that aid with the interpretation of the most parsimonious movement model are provided in Fig. S3 and suggest that, under average conditions, dispersing wild dogs avoided moving through water, woodlands, and areas dominated by humans, but preferred moving across shrublands or grasslands (Fig. 3a). Dispersers realized shorter steps (indicating slower movements) in areas covered by water or woodland, while realizing larger steps in areas dominated by shrubs or grass (Fig. 3a). We found a particularly large effect for the variable LowActivity, suggesting that dispersing wild dogs moved substantially faster during twilight and at night (i.e. between 17:00 and 09:00 o’clock; Fig. 3a). Although dispersers revealed a preference for directional movements (i.e. low turning angles), especially when moving quickly, they did less so in proximity to humans or water, resulting in more tortuous movements in such areas (Fig. 3a).

### Dispersal simulation

Dispersal simulations based on the most parsimonious movement model proved useful for assessing landscape connectivity. Of the 50’000 simulated dispersal trajectories that originated from the main study area, only 4.5% reached a map boundary, suggesting that we successfully mitigated biases from boundary effects. Moreover, our examination of the relative traversal frequency across all checkpoints showed that the relative traversal frequency reached a steady state after 10’500 simulated dispersal trajectories (Fig. S4). Although variability in the relative traversal frequency kept decreasing as we increased the number of simulated dispersers, the marginal benefit of simulating additional trajectories diminished quickly (Fig. S4).

### Heatmap

**Fig. 4:**
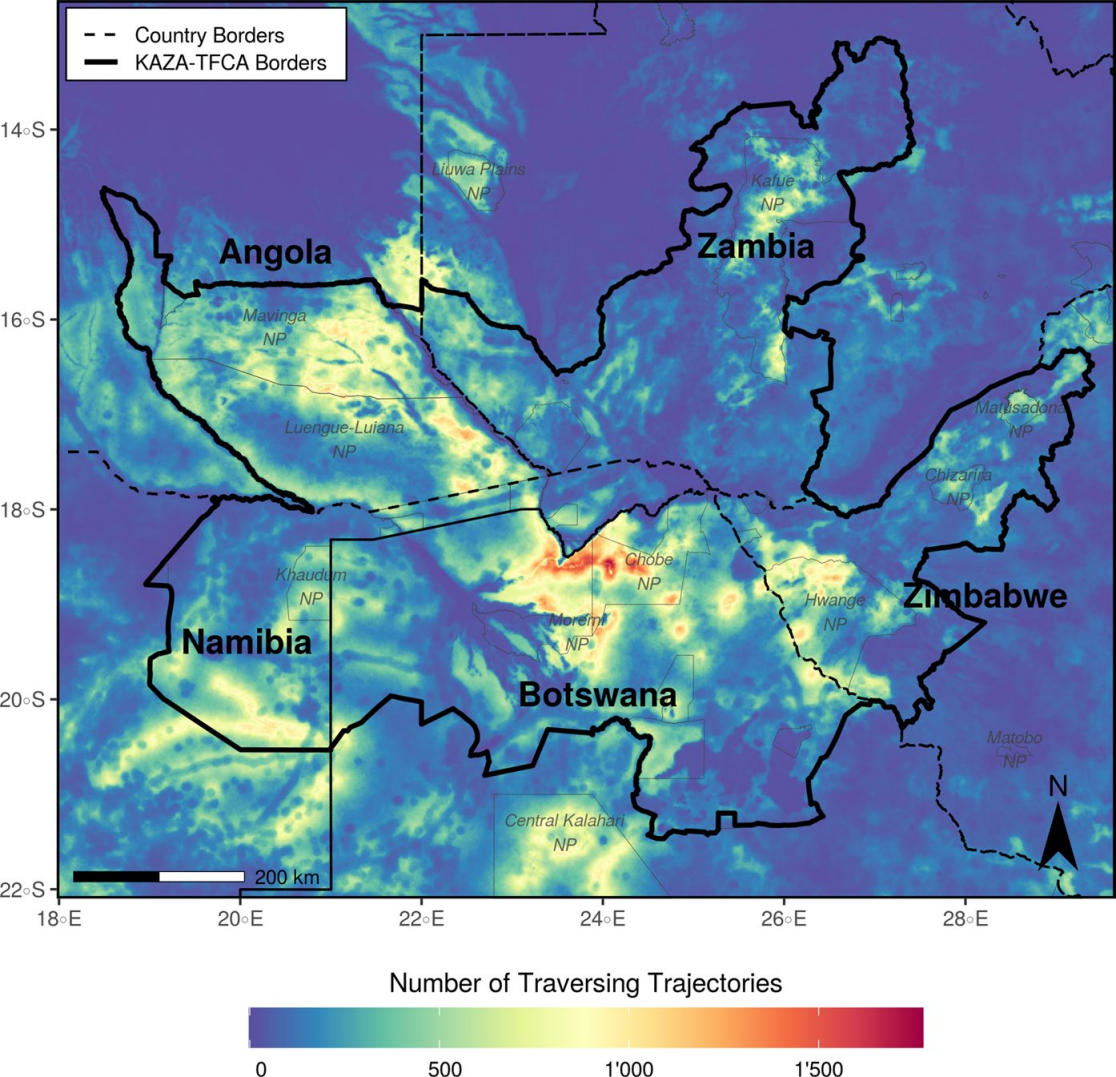
Heatmap showing traversal frequencies of 80’000 simulated dispersers moving 2’000 steps across the KAZA-TFCA. Simulations were based on an integrated step-selection model that we fitted to the movement data of dispersing African wild dogs. To generate the heatmap, we rasterized and tallied all simulated trajectories. Consequently, the map highlights areas that are frequently traversed. For spatial reference we plotted a few selected NPs (dark gray). Additional heatmaps showing the traversal frequency when individuals move fewer than 2’000 steps are provided in Fig. S5

The heatmap (Fig. 4), which resulted from the summation of all simulated dispersal trajectories, allowed us to pinpoint areas that were frequently visited and enabled us to compare areas inside and outside the KAZA-TFCA borders with respect to the intensity at which they were used for dispersal. For instance, we could deduct that areas inside the KAZA-TFCA were frequently traversed by dispersers (median traversal frequency inside KAZA-TFCA = 166, IQR = 274, Fig. S7a), whereas areas beyond the KAZA-TFCA boundary were comparatively rarely visited (median traversal frequency outside KAZA-TFCA = 61, IQR = 133, Fig. S7a). Most notably, the region in northern Botswana south of the Linyanti swamp appeared to serve as highly frequented dispersal hotspot (median traversal frequency = 987, IQR = 558). Aside from revealing movement hotspots, the heatmap also provided information on areas that appeared to hinder movement. For example, extensive water bodies, such as the Okavango Delta, the Makgadikgadi Pan, and the Linyanti swamp, substantially restricted dispersal movements and limited realized connectivity inside the KAZA-TFCA. Similarly, the landscapes of Zambia and Zimbabwe were only rarely used for dispersal, even within the KAZA-TFCA boundaries (Fig. S8a). Despite the fact that the heatmap improved our understanding of the frequency at which areas were traversed by simulated dispersers, it seemed impractical to pinpoint dispersal corridors.

### Betweenness

**Fig. 5:**
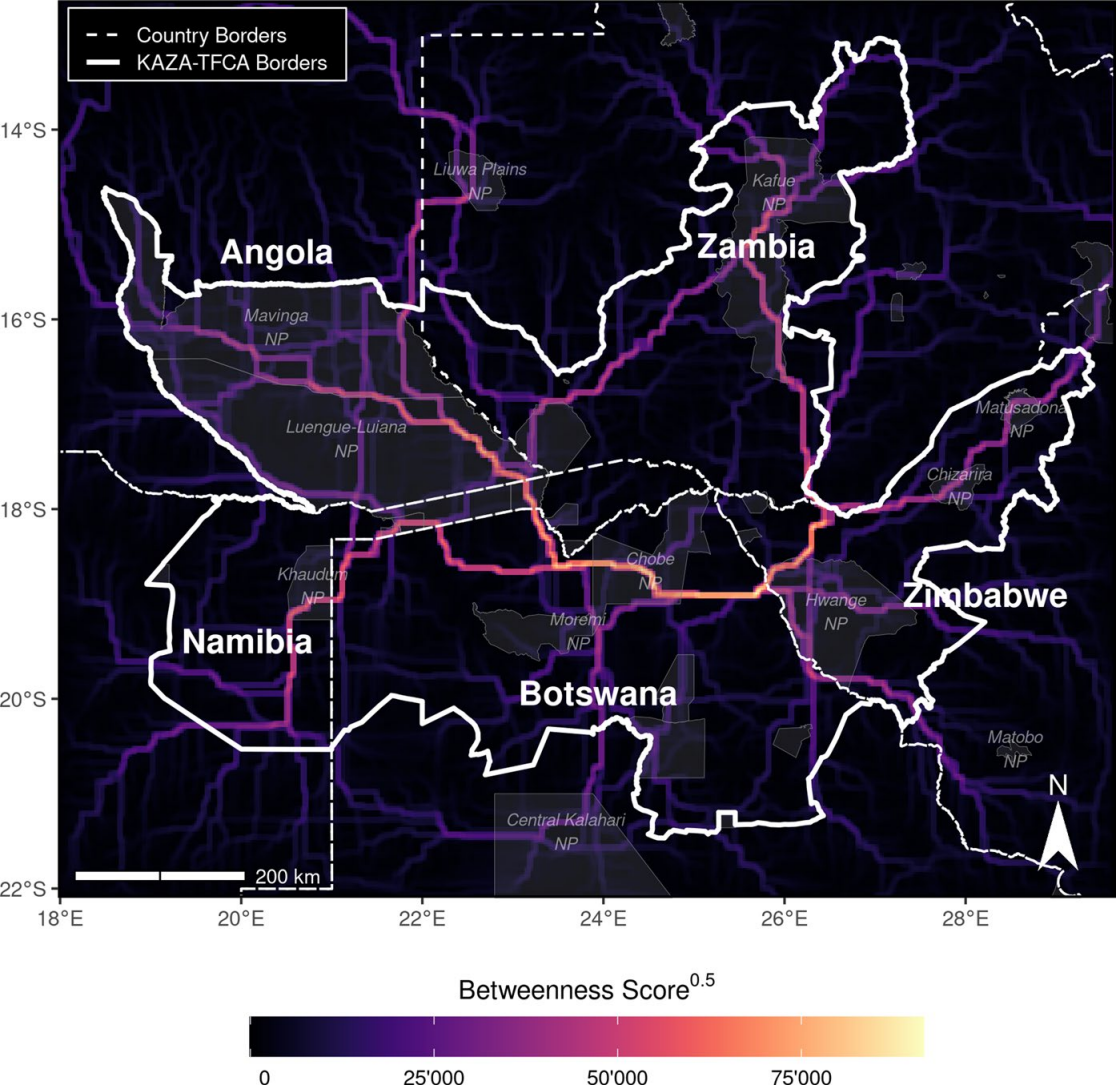
Map of betweenness scores, highlighting distinct dispersal corridors and potential bottlenecks across the extent of the KAZA-TFCA. Betweenness measures the number of shortest paths traversing through each node (raster-cell). Hence, a high betweenness score indicates that the respective area is exceptionally important for connecting different regions in the study area. The metric is therefore useful to pinpoint discrete movement corridors (Bastille-Rousseau et al. 2018). Note that we square-rooted betweenness scores to improve visibility of corridors with comparably low scores. Additional betweenness maps showing betweenness scores when individuals move fewer than 2’000 steps are provided in Fig. S6

The betweenness map (Fig. 5) revealed several distinct dispersal corridors that run across the study area. In comparison to the heatmap, the betweenness map was less biased towards areas with many dispersers and pronounced narrower, more linear routes that were used by simulated individuals to move between regions. Again, northern Botswana emerged as a wild dog dispersal corridor that connected more remote regions in the study area. Towards east, the extension of this corridor ran through Chobe NP into Hwange NP. From there, a further extension connected to Matusadona NP in Zimbabwe. Northwest of the Linyanti ecosystem, a major corridor expanded into Angola, where it split and finally traversed over a long stretch of unprotected area into Zambia’s Kafue NP. Several additional corridors with lower betweenness scores emerged, yet most of them ran within the KAZA-TFCA boundaries (median betweenness inside KAZA-TFCA = 6.947 $\times$ 10$^6$, IQR = 54.311 $\times$ 10$^6$, Fig. S7b). Consequently, only few corridors directly linked the peripheral regions of the KAZA-TFCA and passed through unprotected areas outside its borders (mean betweenness outside KAZA-TFCA = 2.685 $\times$ 10$^6$, IQR = 9.891 $\times$ 10$^6$, Fig. S7b).

### Inter-patch connectivity

**Fig. 6:**
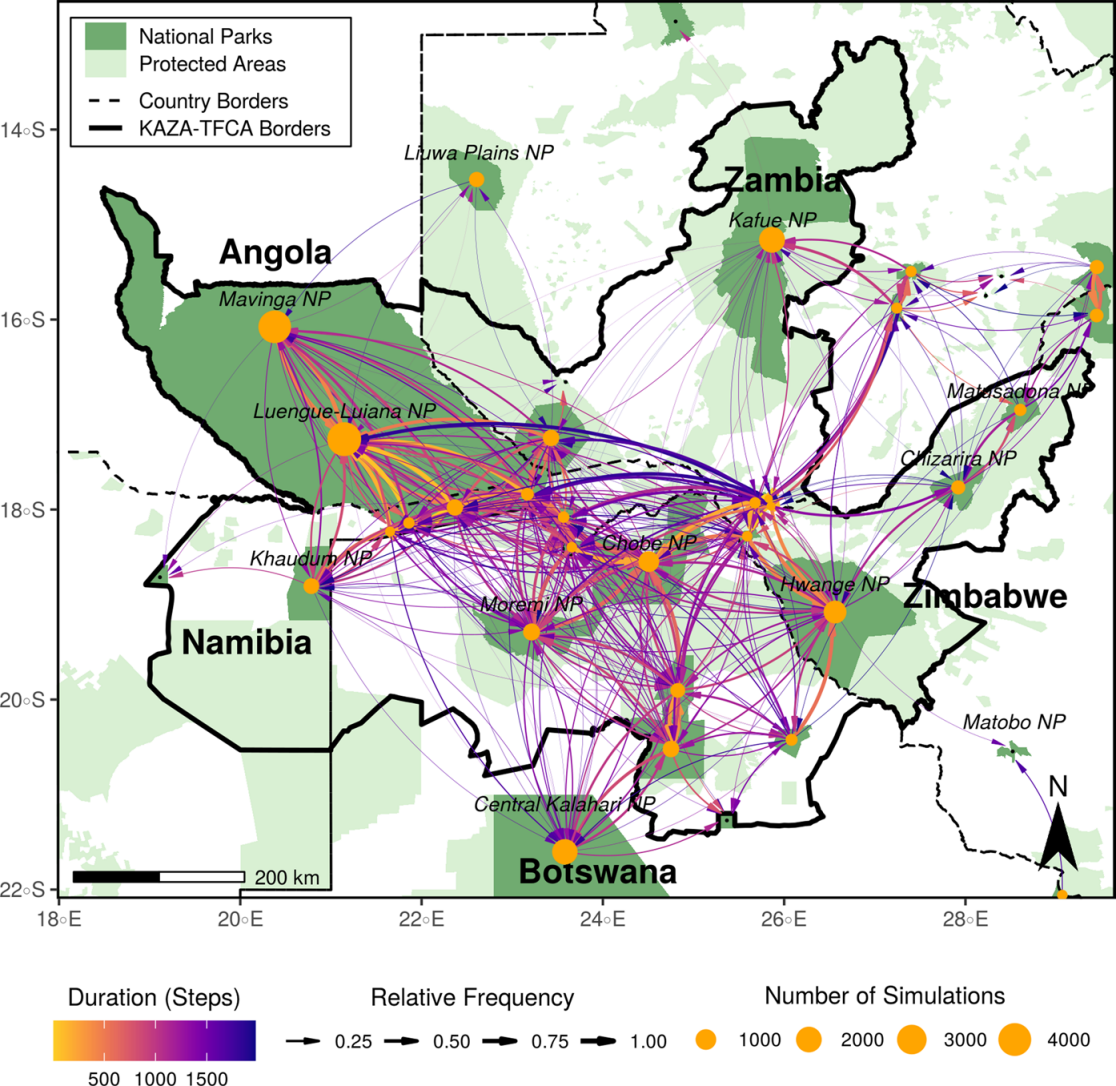
Map of inter-patch connectivity in relation to dispersal duration, highlighting connections between NPs (dark green). Yellow bubbles represent the center of the different NPs and are sized in relation to the number of simulated dispersers originating from each park. Black dots represent NPs that were smaller than 700 km$^2$ and therefore were not used as source areas. Arrows between NPs illustrate between which NPs the simulated dispersers successfully moved and the color of each arrow shows the average number of steps (i.e. 4-hourly movements) that were necessary to realize those connections. Additionally, the line thickness indicates the relative number of dispersers originating from a NP that realized those connections

The inter-patch connectivity map showed that the relative frequency at which simulated dispersal trajectories moved from one patch to another varied, as did the average dispersal duration between patches (Fig. 6). The map thereby completed the picture on connectivity and provided valuable insights into the frequency and duration of connections between patches. For some patches, we also detected imbalances between the number of incoming and outgoing links, hinting at possible source-sink dynamics. From Chobe NP, for instance, 510 individuals reached the Moremi NP, yet the opposite route was only realized by 340 individuals. Relative to the number of simulated individuals, however, these numbers correspond to fractions of 50% and 68%, respectively. Overall, inter-patch connectivity between patches in Angola, Namibia, Botswana, and Zimbabwe appeared to be high; between 54% and 87% of individuals originating from a patch in these countries successfully moved into at least on other patch (Fig. S9a). Conversely, only 19% of the dispersers leaving from a patch in Zambia managed to find their way into some other patch (Fig. S9b). Prior to reaching another patch, individuals from Angola, Namibia, Botswana, Zimbabwe, and Zambia had to move for an average of 630, 640, 940, 1’045, and 890 steps, respectively. Furthermore, it appeared that the corridor previously identified on Fig. 6 between Angola’s NPs and the Kafue NP in Zambia is only rarely realized.

## Discussion

Here, we presented a simple three-step approach to assess landscape connectivity via simulated dispersal trajectories and we demonstrated its application using empirical data from a free-ranging population of African wild dogs. In step one, we used ISSFs to parametrize a fully mechanistic movement model describing how individuals move through the landscape. Aside from rendering habitat preferences, the model also encapsulated movement preferences and potential interactions between movement and habitat preferences. In step two, we employed the movement model and simulated dispersal trajectories across the landscape. In comparison to more traditional connectivity modeling techniques, such simulations require fewer unrealistic assumptions about dispersal and enable the derivation of multiple connectivity metrics. Hence, in step three, we translated the simulated trajectories into three complementary connectivity maps, each emphasizing a different aspect of landscape connectivity (e.g. frequently traversed areas, critical dispersal corridors and bottlenecks, and the presence and intensity of functional links between suitable patches).

Results on the habitat kernel from our model showed that dispersers avoided areas dominated by humans and covered by water, but selected for regions with open grassland in the vicinity to water bodies. This largely complied with previous studies that investigated habitat selection by dispersing wild dogs (Davies-Mostert et al. 2012; Masenga et al. 2016; Woodroffe et al. 2020; O’Neill et al. 2020; Hofmann et al. 2021a). However, instead of merely generating insights on dispersers’ habitat preferences, the ISSF framework also permitted us to model several additional complexities common to dispersal. For instance, by including the interactions cos(ta):sl and cos(ta):log(sl), we could accommodate that dispersers exhibit turning angles that are correlated with step lengths, meaning that turning angles tend to be smaller when individuals move fast. Although similar autocorrelations could be incorporated by sampling step lengths and turning angles from copula probability distributions (Hodel and Fieberg 2022), the ISSF framework allowed us to conveniently model such peculiarities directly in the movement model. While we only considered first order autocorrelation, i.e. correlation between two consecutive steps, higher order autocorrelation is conceivable and may be desirable to model (Dray et al. 2010; McClintock et al. 2012). However, this will require vast amounts of GPS data that are not interrupted by missing fixes; something that is rarely achieved in reality (Graves and Waller 2006). The power and flexibility of ISSFs to model additive effects between habitat and movement covariates (Avgar et al. 2016; Signer et al. 2017) furthermore allowed us to formally capture that dispersing wild dogs move slower and more tortuous in areas covered by water. Such effects may be of limited interest and novelty from a biological perspective, yet they are important to be considered when simulating dispersal, in particular if one is interested in estimating dispersal durations between habitat patches. Overall, the inclusion of interactions between habitat and movement covariates in our movement model lead to a significant improvement in predictive performance compared to an earlier model that omitted such interactions (Hofmann et al. 2021a).

Each of the three connectivity maps derived from simulated dispersal trajectories highlighted a different aspect of landscape connectivity. The heatmap was most suitable for pinpointing frequently traversed areas and showed that an exceptionally large number of dispersers moved through the regions of the Moremi NP and the Chobe NP in northern Botswana. Hofmann et al. (2021a) previously identified the same area as potential dispersal hotspot using LCPA, however, following their analysis it was not clear whether this was the consequence of the central location of the region and connections being enforced between predefined start and endpoints. Contrary to LCPA, a simulation-based approach as presented here does not require predefined endpoints, as endpoints emerge naturally from the simulated dispersal trajectories. This is especially useful for dispersal studies, where known endpoints are usually an unrealistic assumption (Elliot et al. 2014; Abrahms et al. 2017; Cozzi et al. 2020). The fact that the same region was emphasized using vastly different methods to model connectivity thus reinforces our notion that the area is of exceptional importance to dispersing wild dogs. Because simulated individuals are not forced to move towards certain endpoints, a simulation-based approach not only lends itself to study landscape connectivity, but also to uncover potential dispersal traps (Van der Meer et al. 2014) or areas with a high susceptibility for human wildlife conflicts (Cushman et al. 2018). Using independent dispersal data we showed that dispersers indeed followed areas of high predicted connectivity. Importantly, however, these predictions were based on a scenario of a relatively extended flood, which may not have accurately represented environmental conditions for dispersers moving through areas affected by the flood. Accounting for such differences would have improved the predictive performance of our model.

In contrast to the heatmap, the betweenness map emphasized relatively narrow and linear movement routes. It thus facilitated the identification of discrete movement corridors. While in some cases both the heatmap and the betweenness map attributed a high importance to the same areas (e.g. northern Botswana), little consensus was found for other regions. For instance, the stretch of unprotected land between Luengue-Luiana NP in Angola and the Kafue NP in Zambia was characterized by a high betweenness-scores, yet it only received low scores on the heatmap. This is due to the differential way in which the maps view connectivity. While the heatmap attributes a high connectivity to areas that are frequently traversed, it does not distinguish between areas that truly bring individuals into other regions of the study area and regions that lead into ecological traps. The converse is true on the betweenness map, as it strictly highlights regions that promote movement into other areas of the landscape and thus promote gene-flow. However, neither of the two maps provides insights into functional links between distinct habitat patches or how connections depend on the dispersal duration. For this reason, we also produced a map of inter-patch connectivity. This map depicted the frequency at which simulated individuals moved between patches as well as the average dispersal duration (in steps) required to realize them. Calculating dispersal durations was only possible because trajectories were simulated spatially and temporally explicitly, something that is currently unfeasible with LCPA or CT. An explicit representation of time enables answerings questions such as: “*How long will it take a disperser to move from A to B?*” or “*Is it possible for a disperser to move from A to B within X days?*”. Moreover, it yields opportunities to incorporate seasonality and to investigate whether dispersal corridors exist seasonally or all-year round [*dynamic connectivity*; Zeller et al. (2020)]. With LCPA or CT, seasonality can currently only be incorporated through the preparation of multiple permeability surfaces on which the same connectivity model is repeatedly applied (e.g. Osipova et al. 2019). With simulations from ISSFs, in contrast, the environment could change “as the dispersers move”, so that simulated trajectories would dynamically respond to seasonal fluctuations in the environment.

Our approach enabled us to translate a simple set of small-scale behavioral rules into large scale patterns of connectivity, something previously deemed computationally unfeasible, yet critical for linking structural and functional connectivity (Doerr et al. 2011). Structural connectivity focuses purely on the spatial arrangement of suitable habitat in the landscape, whereas functional connectivity also takes into account a species dispersal ability and behavioral response to the landscape (Tischendorf and Fahrig 2000). Functional connectivity is of greater interest to conservation scientists, yet is difficult to quantify (Baguette et al. 2013), which is why structural connectivity often serves as surrogate (Doerr et al. 2011; Fattebert et al. 2015). LCPA and CT incorporate functional aspects of connectivity through the permeability surface, which reflects a species habitat preferences and thus renders behavioral impacts of the lanscape on the focal species. Aside from rendering habitat preferences, our model also integrates peculiarities of the focal species movement behavior, thus adding further insights on functional connectivity. In addition, we successfully used independent dispersal data to prove that our predictions of connectiviy aligned with observed functional connectivity patterns.

Despite the many benefits and great flexibility offered by simulations from ISSFs, one must also be aware of the associated limitations. For example, while our approach of simulating dispersal proved usedful to assess landscape connectivity, it was computationally costly. Simulating 80’000 dispersal trajectories for 2’000 steps across the KAZA-TFCA required five days of computation on a regular desktop machine (AMD Ryzen 7 2700X octa-core processor with 3.6 GHz, 64 GB of RAM). The long simulation time was primarily caused by the massive extent of the study area considered (ca. 1.3 Mio km$^2$), the large number of simulated trajectories, and the fact that we extracted covariates along each step, rather than just at their start or endpoints. Most connectivity studies focus on smaller study areas (e.g. Kanagaraj et al. 2013; Clark et al. 2015; McClure et al. 2016; Abrahms et al. 2017; Zeller et al. 2020) and will therefore require fewer simulations and achieve faster simulation times (given the same spatial resolution). We also believe that fewer simulated trajectories will often suffice, as the relative traversal frequency by simulated trajectories through randomly placed checkpoints across our study area converged already after 10’500 runs. The exact number of required simulations to achieve reliable estimates of connectivity will, of course, vary depending on the structure of the landscape and the dispersal capabilities of the focal species (Gustafson and Gardner 1996). For species that disperse short distances through homogeneous environments, few simulations may suffice to gauge connectivity, whereas for species that disperse over long distances through heterogeneous habitats, a large number of simulations will be required to sufficiently explore the spectrum of possible routes. Finally, it may often suffice to extract covariates at each step’s start or endpoints, thus considerably speeding up simulation times (Signer et al. 2017).

Aside from the computational requirements, simulations further entail several non-trivial but important modeling decisions. On four such decisions we would like to further elaborate: (1) the number of simulated individuals, (2) the location of source points, (3) the simulated dispersal duration, and (4) the behavior at map boundaries.

1. When simulating dispersal trajectories, the modeler needs to decide on the number of simulated individuals. A higher number is always desirable, as each additional trajectory provides information about landscape connectivity. However, each additional simulation imposes computational costs, so a trade-off needs to be managed. Signer et al. (2017) proposed to handle the trade-off by simulating additional individuals only until the metrics of interest converge towards a steady state. Here, we used the relative traversal frequency as target metric and found that it converged already after 10’500 simulated individuals. The exact number of required individuals might, however, vary depending on the employed target metric and the anticipated connectivity map. More sophisticated target metrics than the relative traversal frequency, preferably tailored to different connectivity maps, need to be developed in the future.
2. To initiate dispersers, a modeler needs to provide a set of source points at which the virtual dispersers are released. We placed source points within protected areas large enough to sustain viable wild dog populations, implicitly assuming that wild dogs primarily survive in large, formally protected areas (Davies-Mostert et al. 2012; Woodroffe and Sillero-Zubiri 2012; Van der Meer et al. 2014). Moreover, we lacked precise knowledge about the distribution and abundance of wild dogs across protected areas, so we placed source points randomly within them. In cases where more detailed data about the distribution and abundance of the focal species are available, source points could be distributed accordingly. Alternatively, source points could be distributed homogeneously but later be weighted when computing connectivity metrics. In any case, the challenge of selecting meaningful source points is not unique to individual-based simulations but also applies to LCPA and CT.
3. The use of ISSFs to simulate dispersers requires deciding on the number of simulated steps (i.e. the simulated dispersal durations). If sufficient dispersal data of the focal species has been collected, dispersal durations could be sampled from observed dispersal events or from parametric distributions fit to observed data. Due to the low number of observed dispersal events, we opted against this solution and instead simulated all individuals for 2’000 steps, which was at the upper end of observed dispersal durations in African wild dogs (Davies-Mostert et al. 2012; Masenga et al. 2016; Cozzi et al. 2020; Hofmann et al. 2021a). This approach had the advantage that it allowed us to systematically shorten the simulated trajectories after their simulation and thereby to investigate the sensitivity of our results with respect to exact dispersal durations (Figs. S5 and S6).
4. Unless simulated dispersal trajectories are strongly drawn towards a point of attraction inside the study area (e.g. Signer et al. 2017), some trajectories will inevitably approach one of the map boundaries. In this case, one or more of the generated random steps might leave the study area, making it impossible to compute a selection score. A possible solution is to simply terminate the simulation of the affected trajectory, assuming that the simulated individual has left the study area. However, this approach might produce ambiguous results in cases where many individuals are released near map borders, especially because already a single random step leaving the study area will break the simulation, thus resulting in biased connectivity estimates along map borders. Rather than breaking the simulation, we created a buffer zone (Koen et al. 2010) and resampled random steps until they fully lied within the study area. This proved to be an effective solution to overcome problems with boundary effects.

In summary, we proposed and applied a simple three-step approach that relies on ISSF-analysis and enables the simulation of dispersal trajectories and the assessment of landscape connectivity. The proposed approach overcomes several of the conceptual shortcomings inherent to LCPA and CT, such as the assumption of known endpoints, and provides a highly flexible tool for investigating connectivity. Moreover, the simulation of dispersal opens up new avenues for incorporating interactions between habitat and movement covariates and provides the foundation for a rich suite of complementary connectivity measures. With this work, we hope to have sparked interest in the application, optimization, or creation of methods to investigate dispersal and connectivity via individual-based simulations, while at the same time stressing some of the non-trivial modeling decisions involved. We also hope to provide a useful framework that helps unifying and streamlining the application of individual-based simulations for assessing landscape connectivity.

## Supplementary Information

Below is the link to the electronic supplementary material.

Supplementary file 1 (pdf 15327 KB)

## Data Availability

GPS movement data of dispersing wild dogs is available on dryad (Hofmann et al. 2021b). Access to R-scripts that exemplify the application of the proposed approach using simulated data, as well as all codes required to reproduce the African wild dog case study are provided through Github (https://github.com/DavidDHofmann/DispersalSimulation).
