# Supplementary material for: A three-step approach for assessing landscape connectivity via simulated dispersal: African wild dog case study
Source: Landsc Ecol. 2023 Feb 17;38(4):981–98. doi: 10.1007/s10980-023-01602-4 (PMC10020313; doi:10.1007/s10980-023-01602-4)
Supplement: Supplementary file 1 — (pdf 15327 KB) [file 10980_2023_1602_MOESM1_ESM.pdf]

Appendix

A Three-Step Approach for Assessing Landscape Connectivity via Simulated Dispersal: African Wild Dog Case Study

David D. Hofmann[1,2,§] Gabriele Cozzi[1,2] John W. McNutt[2]
Arpat Ozgul[1] Dominik M. Behr[1,2]

December 13, 2022

[1] Department of Evolutionary Biology and Environmental Studies, University of Zurich, Winterthurerstarsse 190, 8057 Zurich, Switzerland.

[2] Botswana Predator Conservation Program, Wild Entrust, Private Bag 13, Maun, Botswana.

[§] Corresponding author (david.hofmann2@uzh.ch)

Running Title: Simulating Wild Dog Dispersal Trajectories to Assess Landscape Connectivity

Keywords: dispersal, simulation, movement, integrated step selection function, Kavango-Zambezi Transfrontier Conservation Area, landscape connectivity, Lycaon pictus

A.1 Candidate Interactions

We started with the base model developed by Hofmann et al. (2021) and incrementally increased model complexity by adding all possible two-way interactions between habitat covariates and movement covariates. For instance, for the covariate Water, we proposed the interactions Water:sl, Water:log(sl), and Water:cos(ta). Besides these interactions, we also allowed for correlations between turning angles and step lengths by proposing the interactions sl:cos(ta) and log(sl):cos(ta). Furthermore, we formed the interactions sl:LowActivity and log(sl):LowActivity to render that step lengths are likely to be shorter during periods of inactivity.

A.2 K-Fold Cross Validation Procedure

We validated the predictive power of the most parsimonious movement model using k-fold cross-validation for case-control studies Fortin et al. (2009). Specifically, we randomly assigned 80% of the strata to a training set and the remaining 20% to a testing set. Using the training set, we parametrized a movement model and predicted selection scores $w(x)$ for all steps in the testing set. Within each stratum, we then assigned ranks 1-25 to each step based on predicted selection scores, so that rank 1 was given to the step with the highest score $w(x)$. Within each strata, we determined the realized step's rank and calculated rank frequencies of realized steps across all strata. Finally, we computed Spearman's rank correlation between ranks and associated frequencies $r_{s,realized}$. We replicated this procedure 100 times and computed the mean correlation coefficient ($\bar{r}_{s,realized}$), as well as its 95% confidence interval across all replicates. For comparison, we repeated the same procedure 100 times assuming random preferences. Random preferences were implemented by discarding the realized step from all strata and identifying the rank of a random step in each stratum. Again, we calculated Spearman's rank correlation coefficient ($r_{s,random}$), its mean across repetitions ($\bar{r}_{s,random}$), and its 95% confidence interval. Ultimately, this validation proves a significant prediction in case the confidence intervals of $\bar{r}_{s,realized}$ and $\bar{r}_{s,random}$ do not overlap (Fortin et al., 2009).

A.3 Source Areas & Points

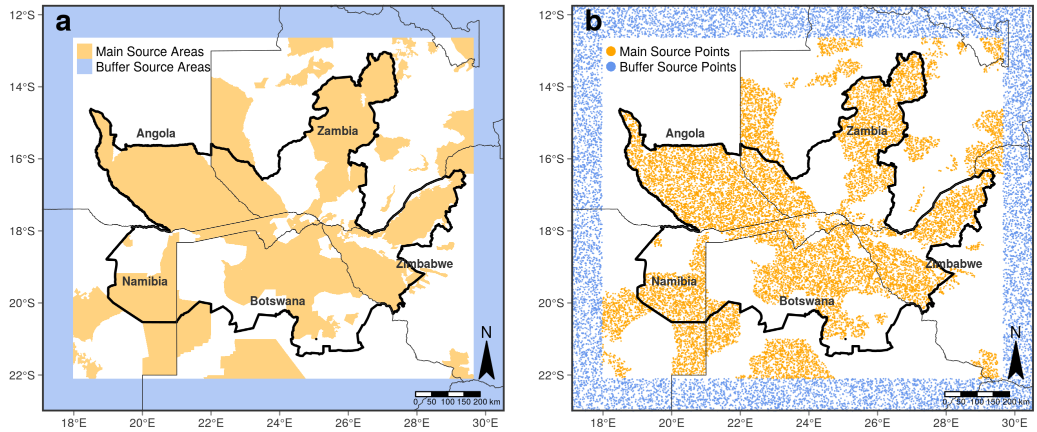

Figure S1: (a) Different source areas from which we released virtual dispersers. We only considered contiguous protected areas (national parks, game reserves, and forest reserves) that were larger than 700 km^2. This size corresponds to the average home range requirement for viable wild dog populations (Pomilia et al., 2015). To render potential immigrants into the study system, we also initiated dispersers within a buffer zone (blue) surrounding the main study area. (b) Source points from which dipsersers were released. 50'000 dispersers were released within the main study area (green dots) and another 30'000 dispersers within the virtual buffer (blue dots).

A.4 Betweenness Maps

To generate betweenness maps, we first overlaid the study area with a regular grid with a resolution of 2.5 km x 2.5 km per grid cell. The grid cells were labelled with unique numbers, which allowed us to then generate a "visitation history" for each simulated trajectory, showing through which grid cells each simulated invidual moved. Based on this history, we tallied the number of transitions that occured from one cell to another. Ultimately, the so generated list of transitions served to generate a network and to compute betweenness scores. The resolution of the overlaid grid thus dictates the resolution at which betweenness can be mapped and has important implications for the way in which cell-transitions need to be determined. In the simplest case, cell-transitions can be determined by only looking at the start and endpoint of each step. If it ends in a different grid-cell than it started, a transition has occured. This approach is computationally simple as it only requires a point extraction (at the start and endpoint of each step). However, relying on a point extraction has the disadvantage that transitions between non-adjacent cells can occur if a single step stretches across multiple grid-cells. Especially when the resolution of the grid is high, this problem becomes more pertinent. As an alternative, one could not only consider the start and endpoint of each step, but the entire line along the step, i.e. conduct a line extraction. However, this requires that extracted values (i.e. grid-cell labels) are ordered in accordance to the direction of movement. In R, such an extraction can be achieved e.g. using `raster::extract(raster, line, along = T)`, which is computationally very demanding and therefore often an unviable solution. Here, we employed yet another approach and generated interpolated coordinates along each simulated step. We then determined cell transition using those interpolated coordinates. Since this approach still uses a point extraction, it is considerably faster while still enabling to detect cell-transitions at greater resolution. In Figure S2, we use simulated movement paths to compare betweenness maps that are based on a (1) point extraction, a (2) line extraction, and a (3) point extraction with interpolated coordinates. As you can see, the approach using interpolated coordinates approximates the results from the line extraction very closely, even at higher resolutions. A detailed discussion about this is can also be found in (Bastille-Rousseau et al., 2018).

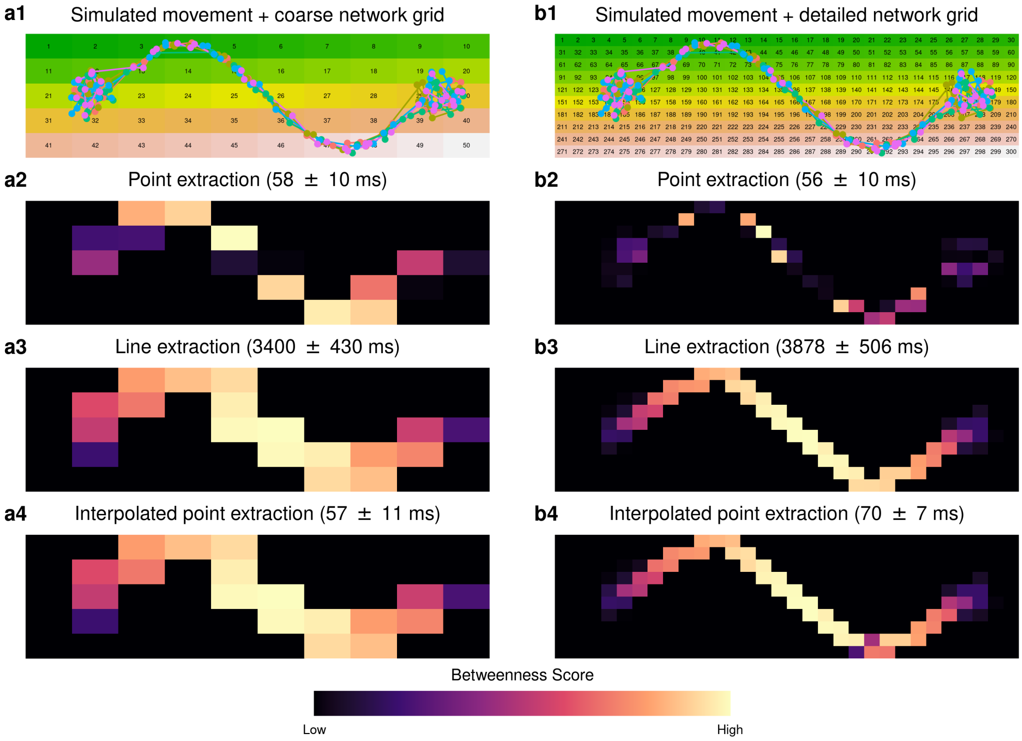

Figure S2: We simulated five trajectories where individuals moved through a narrow corridor. We then overlaid those simulations with a coarse (a1) and detailed (b1) grid that was used to compute betweenness scores. This required us to determine cell-transitions, which we did using three competing approaches. Once using a point extraction (a2 and b2), once using a line extraction (a3 and b3), and once using a point extraction with interpolated coordinates (a4 and b4). In brackets are the average computation times ± SD as computed from 10 repetitions.

A.5 Model Selection Results

Table S1: Results from the forward model selection procedure based on Akaike's Information Criterion (AIC; Burnham and Anderson, 2002). The model in the top row was the model that we used to simulate movement of dispersers. The base model upon which we based our movement model is depicted in the last row and was originally presented in Hofmann et al. (2021). We omitted all models with an AIC weight of zero from the table.

Covariates	AIC	ΔAIC	Weight	LogLik
**Base Model** + sl:LA + WA:sl + log(sl):cos(ta) + DTW:cos(ta) + WO:sl + HI:cos(ta) + SH:sl + DTW:sl + sl:cos(ta)	89392.88	0.00	0.15	-44670.44
**Base Model** + sl:LA + WA:sl + log(sl):cos(ta) + DTW:cos(ta) + WO:sl + HI:cos(ta) + SH:sl + DTW:sl + sl:cos(ta) + SH:log(sl)	89393.92	1.04	0.09	-44669.96
**Base Model** + sl:LA + WA:sl + log(sl):cos(ta) + DTW:cos(ta) + WO:sl + HI:cos(ta) + SH:sl + DTW:sl + sl:cos(ta) + DTW:log(sl)	89394.13	1.25	0.08	-44670.06
**Base Model** + sl:LA + WA:sl + log(sl):cos(ta) + DTW:cos(ta) + WO:sl + HI:cos(ta) + SH:sl + DTW:sl + sl:cos(ta) + WO:log(sl)	89394.25	1.37	0.08	-44670.13
**Base Model** + sl:LA + WA:sl + log(sl):cos(ta) + DTW:cos(ta) + WO:sl + HI:cos(ta) + SH:sl + DTW:sl	89394.36	1.48	0.07	-44672.18
**Base Model** + sl:LA + WA:sl + log(sl):cos(ta) + DTW:cos(ta) + WO:sl + HI:cos(ta) + SH:sl + DTW:sl + sl:cos(ta) + log(sl):LA	89394.44	1.56	0.07	-44670.22
**Base Model** + sl:LA + WA:sl + log(sl):cos(ta) + DTW:cos(ta) + WO:sl + HI:cos(ta) + SH:sl + DTW:sl + sl:cos(ta) + HI:sl	89394.56	1.68	0.07	-44670.28
**Base Model** + sl:LA + WA:sl + log(sl):cos(ta) + DTW:cos(ta) + WO:sl + HI:cos(ta) + SH:sl + DTW:sl + sl:cos(ta) + WA:log(sl)	89394.57	1.69	0.07	-44670.29
**Base Model** + sl:LA + WA:sl + log(sl):cos(ta) + DTW:cos(ta) + WO:sl + HI:cos(ta) + SH:sl + DTW:sl + sl:cos(ta) + WO:cos(ta)	89394.59	1.71	0.07	-44670.30
**Base Model** + sl:LA + WA:sl + log(sl):cos(ta) + DTW:cos(ta) + WO:sl + HI:cos(ta) + SH:sl + DTW:sl + sl:cos(ta) + WA:cos(ta)	89394.63	1.75	0.06	-44670.31
**Base Model** + sl:LA + WA:sl + log(sl):cos(ta) + DTW:cos(ta) + WO:sl + HI:cos(ta) + SH:sl + sl:cos(ta)	89394.68	1.80	0.06	-44672.34
**Base Model** + sl:LA + WA:sl + log(sl):cos(ta) + DTW:cos(ta) + WO:sl + HI:cos(ta) + SH:sl + DTW:sl + sl:cos(ta) + HI:log(sl)	89394.69	1.81	0.06	-44670.35
**Base Model** + sl:LA + WA:sl + log(sl):cos(ta) + DTW:cos(ta) + WO:sl + HI:cos(ta) + SH:sl + DTW:sl + sl:cos(ta) + SH:cos(ta)	89394.84	1.96	0.06	-44670.42
⋮	⋮	⋮	⋮	⋮
**Base Model**: cos(ta) + sl + log(sl) + WA + WO + DTW + HI + SH	90091.40	787.67	0.00	-45030.70

Note: ta = Turning Angle, sl = Step Length, LA = Low Activity, WA = Water, DTW = Distance To Water, SH = Shrubs/Grassland, WO = Woodland, HI = Human Influence.

A.6 Movement Model

Table S2: Most parsimonious movement model for dispersing wild dogs. The model consists of a movement kernel, a habitat kernel, and their interactions. The movement kernel describes preferences with regards to movement behavior, whereas the habitat kernel describes preferences with respect to habitat conditions. Interactions between the two kernels indicate that movement preferences are contingent on habitat conditions. Note that all covariates were standardized to a mean of zero and standard deviation of 1. Plots to aid with the interpretation of this model are given in Appendix S2.

Kernel	Covariate	Coefficient	SE	p-value	Sign.
Habitat Kernel	Water	-0.546	0.112	< 0.001	***
	DistanceToWater $^{0.5}$	-0.390	0.231	0.092	*
	Woodland	-0.364	0.086	< 0.001	***
	Shrubs/Grassland	0.288	0.092	0.002	***
	HumanInfluence	-0.535	0.229	0.019	**
Movement Kernel	sl	0.075	0.037	0.042	**
	cos(ta)	0.105	0.031	0.001	***
	log(sl)	0.146	0.051	0.004	***
	cos(ta) : sl	0.049	0.026	0.064	*
	cos(ta) : log(sl)	0.076	0.026	0.003	***
	sl : LowActivity	-0.917	0.113	< 0.001	***
Interactions	sl : Water	-0.305	0.076	< 0.001	***
	sl : Woodland	-0.089	0.039	0.023	**
	sl : Shrubs/Grassland	0.124	0.058	0.032	**
	sl : DistanceToWater $^{0.5}$	-0.058	0.031	0.056	*
	cos(ta) : HumanInfluence	-0.040	0.022	0.070	*
	cos(ta) : DistanceToWater $^{0.5}$	0.063	0.026	0.017	**

Significance codes: * $p < 0.10$ ** $p < 0.05$ *** $p < 0.01$

A.7 Movement Model Interpretation

To ease with the interpretation of the most parsimonious movement model, we followed recommendations published in Fieberg et al. (2021) and produced a series of plots highlighting how the habitat and movement kernel depended on covariate values (Figure S3). To visualize the movement kernel and its interactions with other covariates, we used model estimates and updated our tentative distribution parameters for turning angles (von Mises distribution with concentration $\kappa = 0$) and step lengths (gamma distribution with scale $\theta =$ 6'308 and shape $k = 0.37$) by applying the function `update_vonmises()` from the R-package `amt` (Signer et al., 2019). This allowed us to compute probability densities of turning angles and step lengths under varying values of the associated covariates, while holding all other covariates constant (Figure S3, a1-a8). Moreover, we investigated the habitat kernel by computing relative selection strengths (RSS) between a set of steps where values of the covariate of interest was varied to a reference step where the covariate value was fixed to its centered value. To illustrate model uncertainty, we also generated large-sample confidence intervals using standard errors associated with each model estimate (Figure S3, b1-b5).

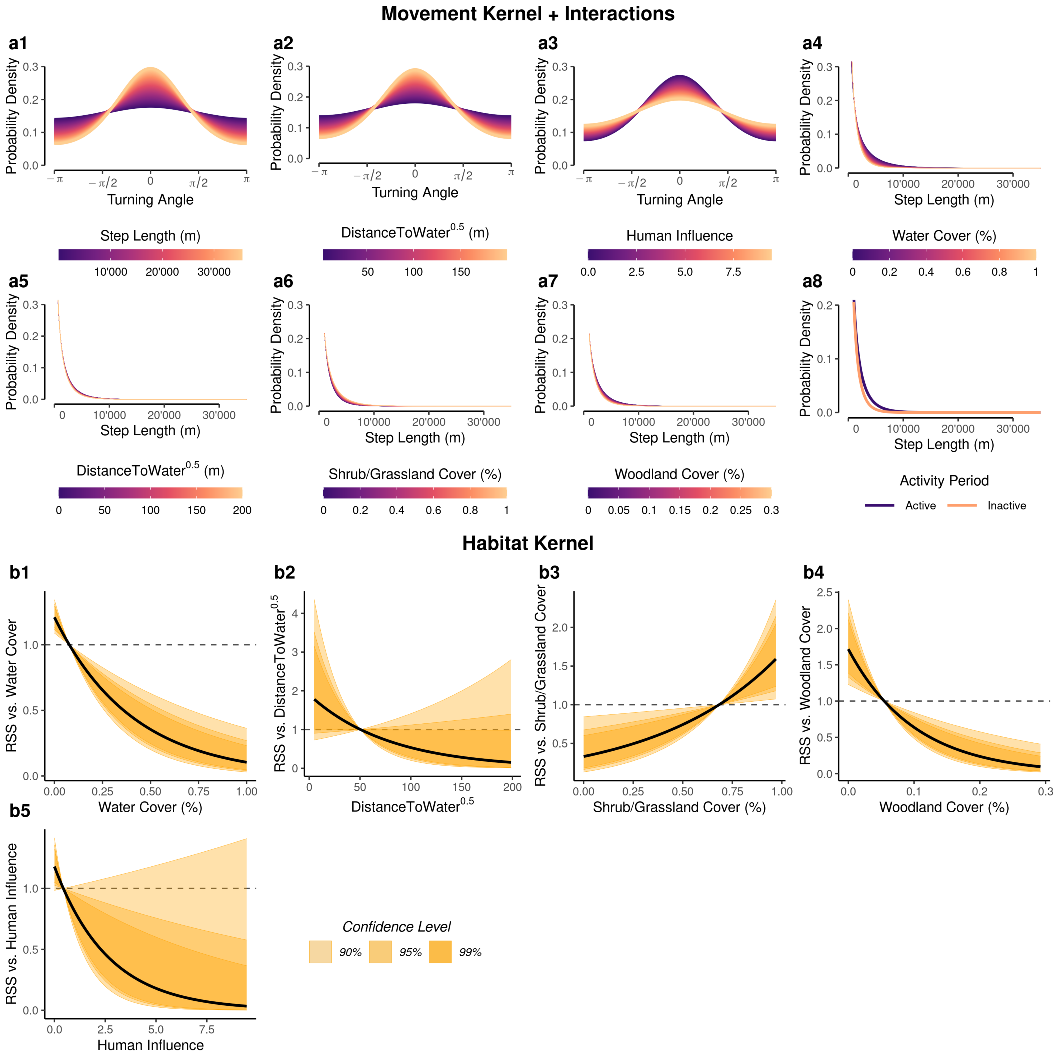

Figure S3: Auxiliary plots that help with the interpretation of the most parsimonious movement model from Table S1. The plots were generated following recommendations reported in Fieberg et al. (2021). Subplots a1 to a8 highlight dispersing wild dogs' movement kernel and indicate how the kernel is influenced by interactions with other covariates. Subplots b1 to b5 depict results from dispersing wild dogs' habitat kernel and highlight differences in predicted relative selection scores (RSS) when varying values of the covariate of interest. For each covariate, predictions were made on the range of values that was observed in the real data, assuming that all other covariates were centered and that steps were realized during periods of "high" wild dog activity. Plot a1, for example, can be interpreted as follows: the probability of realizing a step with a low turning angle is much higher when the corresponding step is large. Moreover, b1 can be interpreted as follows: relative probability of using a step decreases as the amount of water cover along the step increases.

A.8 Convergence

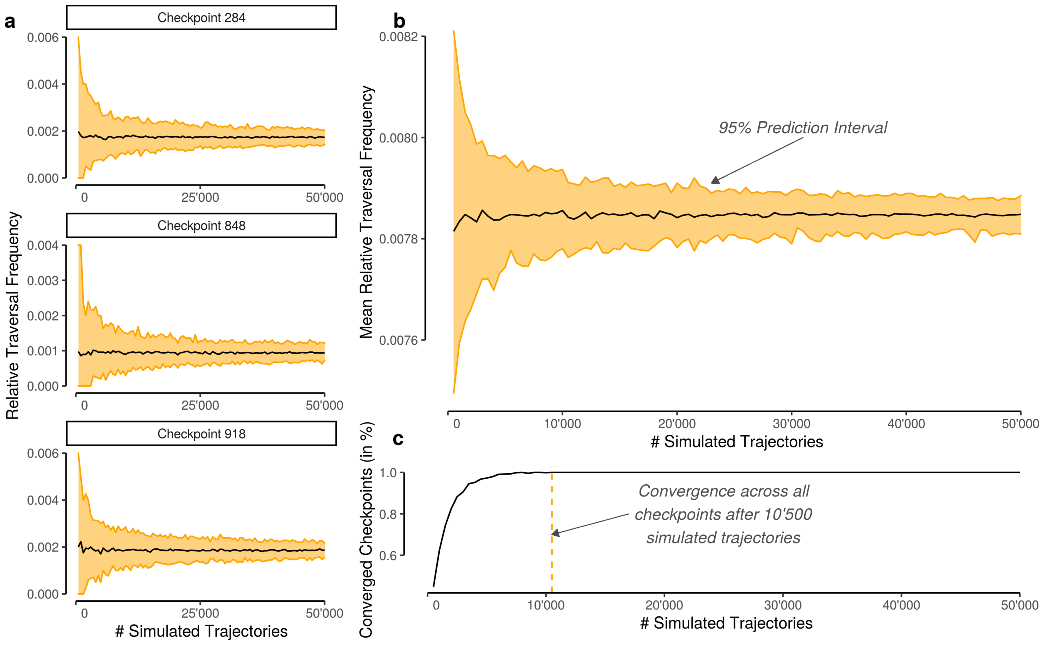

Figure S4: Relative traversal frequency through 1'000 checkpoints (5 km x 5 km) distributed randomly across the study area. The relative traversal frequency is plotted against the number of simulated individuals to visualize how quickly the metric converges to a steady state. (a) Replicated (100 times) relative traversal frequencies across three randomly chosen checkpoints as well as the corresponding 95% prediction interval (PI). (b) Averaged relative traversal frequency across all checkpoints and replicates including a 95% PI. (c) Width of the PI in relation to the number of simulated dispersers.

A.9 Heatmaps in Relation to the Number of Simulated Steps

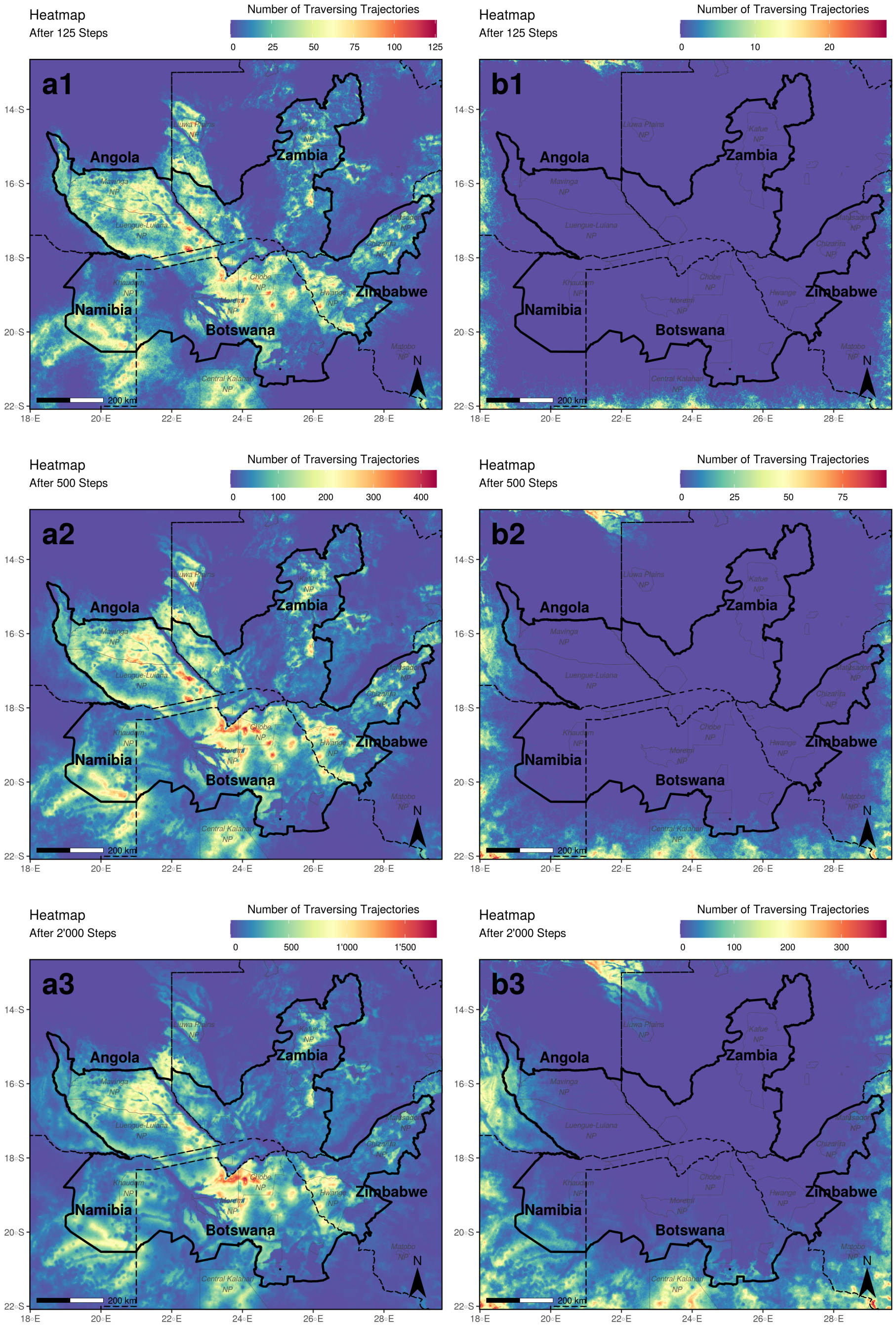

Figure S5: Heatmaps produced when considering 125, 500, and 2000 simulated steps, respectively. The left panel (a1, a2, a3) was generated based on simulations initiated within the main study area, the right panel (b1, b2, b3) was generated based on simulations initiated within the buffer area. To produce the heatmap presented in the main manuscript (Figure 5), we tallied the values from maps a3 and b3.

A.10 Betweenness Maps in Relation to the Number of Simulated Steps

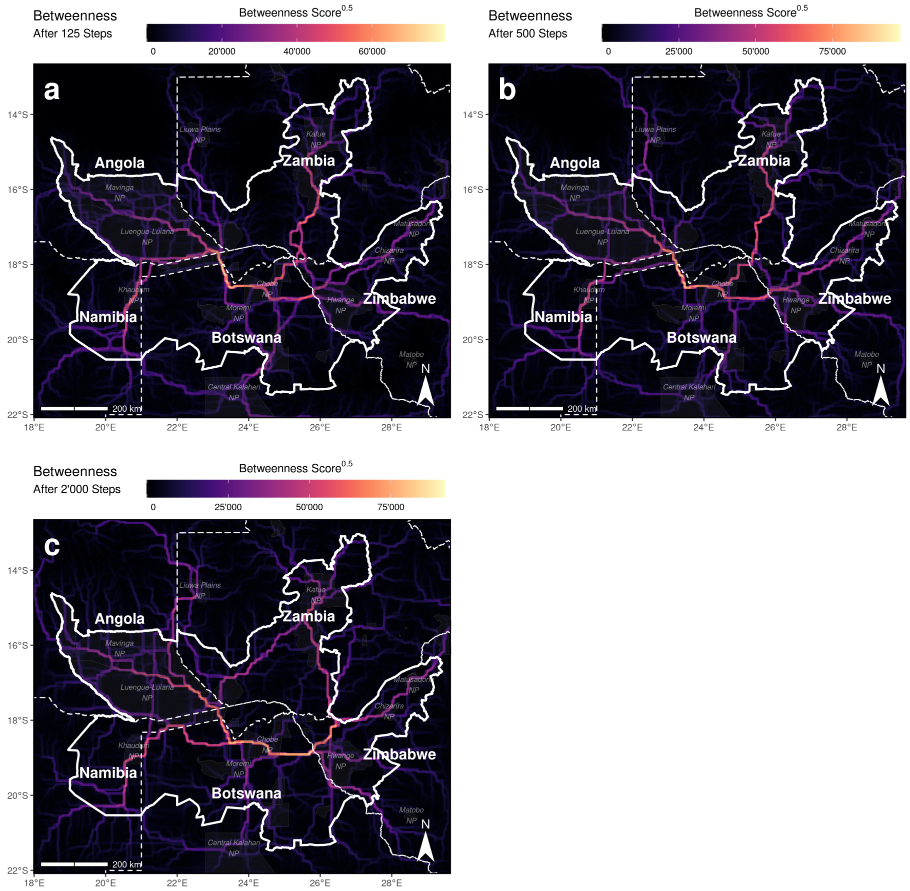

Figure S6: Maps of betweenness scores produced when considering (a) 125, (b) 500, (c) and 2000 simulated steps, respectively. A high betweenness score indicates that the respective area has a high importance for linking other regions in the study area.

A.11 Comparison of Traversal Frequencies and Betweenness Scores Inside and Outside KAZA-TFCA

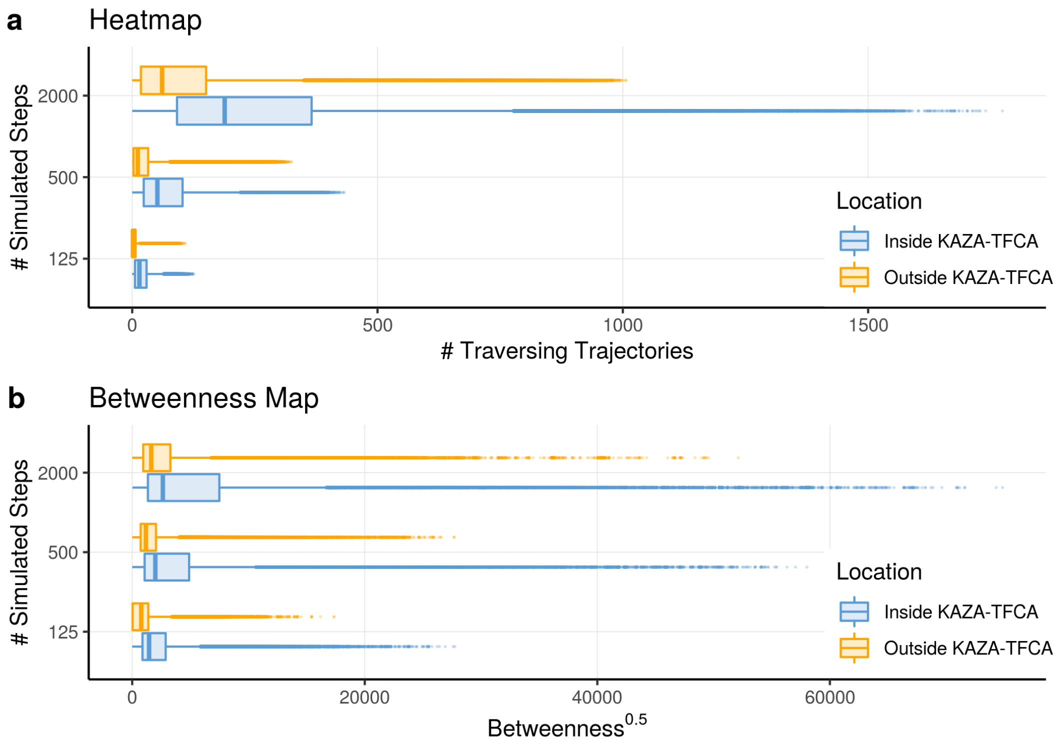

Figure S7: Comparison of values from the heatmap and betweenness map inside (blue) and outside (orange) the KAZA-TFCA borders.

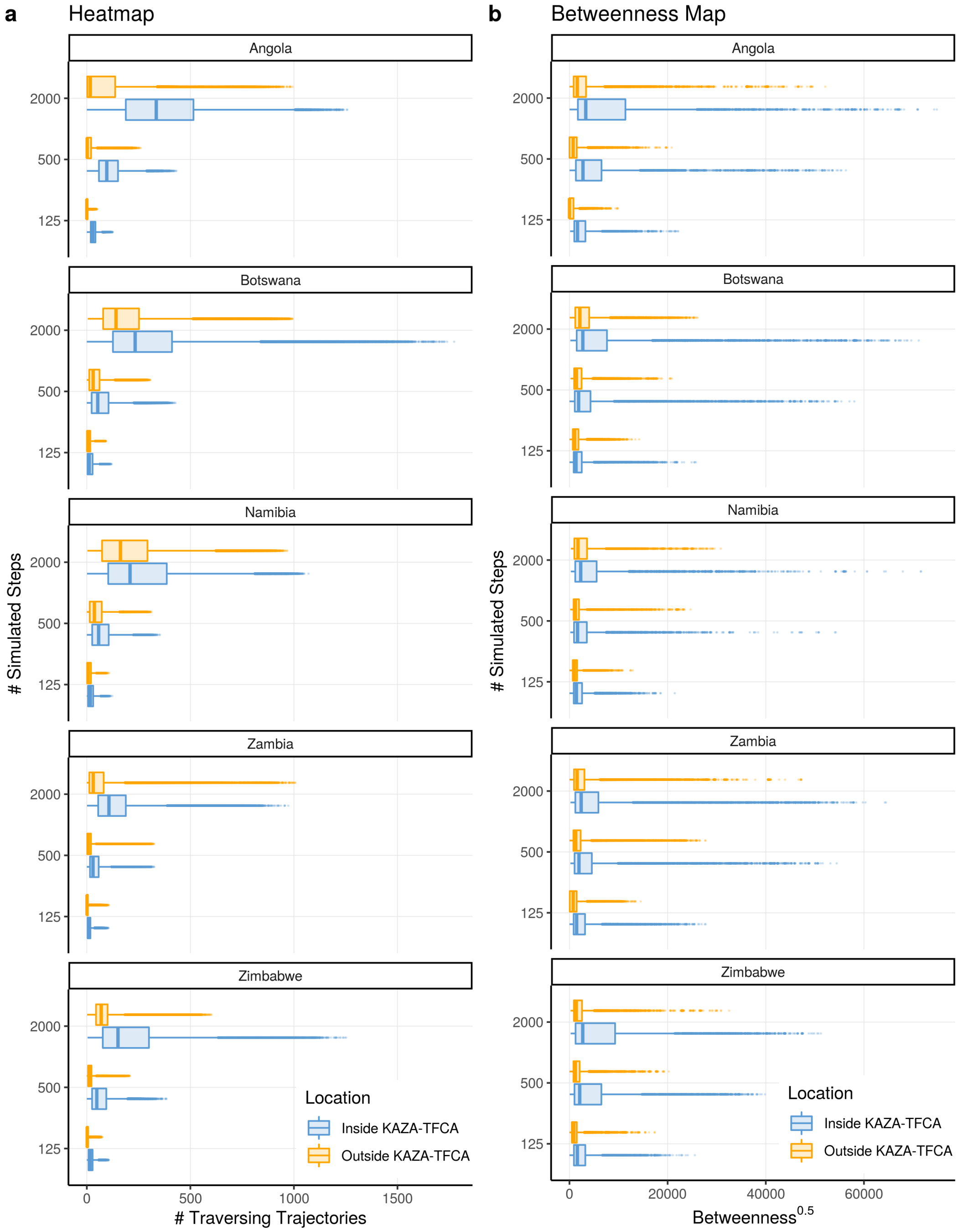

Figure S8: Comparison of values from the heatmap and betweenness map inside (blue) and outside (orange) the KAZA-TFCA borders within different countries.

A.12 Dispersal into other National Parks

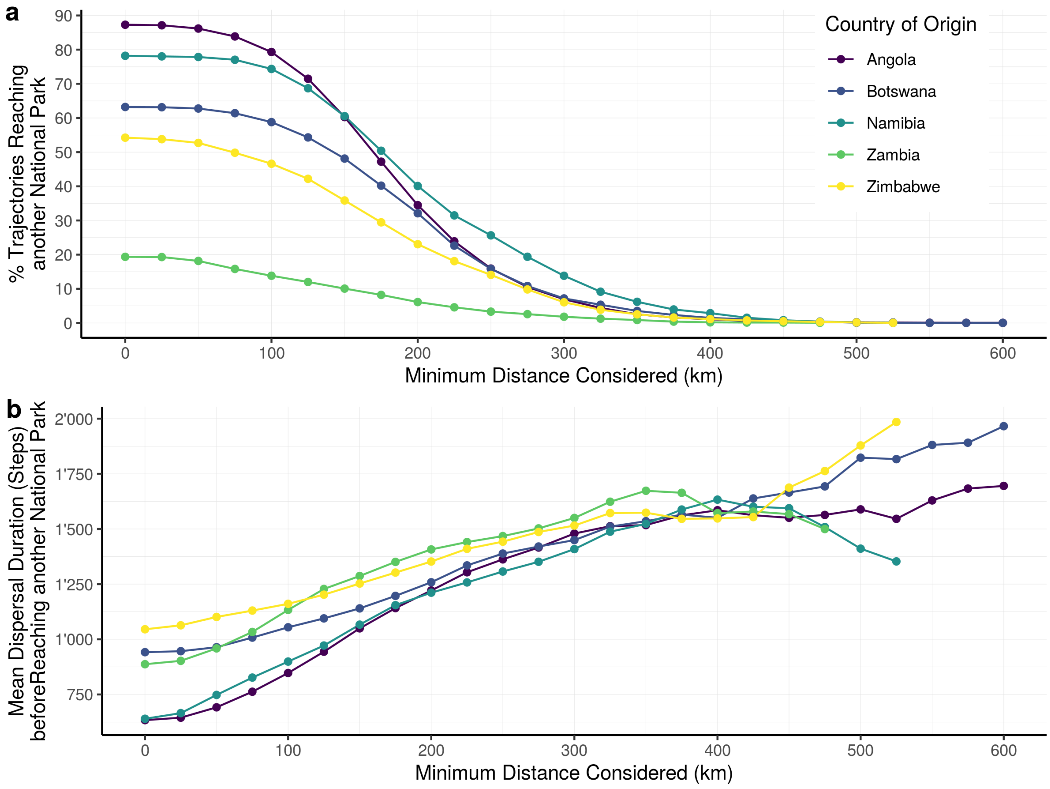

Figure S9: Relative number of simulated dispersal trajectories that successfully moved from one national park into another that is at least as far away as indicated on the x-axis. Percentages are given in relation to the number of simulated individuals from the national parks in the respective countries. For example, over 85% of all individuals originating from a national park in Angola moved from their natal national park into another one. However, the percentage gradually decreases as only national parks at higher euclidean distances are considered.

References

Bastille-Rousseau, G., Douglas-Hamilton, I., Blake, S., Northrup, J. M., and Wittemyer, G. (2018). Applying Network Theory to Animal Movements to Identify Properties of Landscape Space Use. *Ecological Applications*, 28(3):854–864.

Burnham, K. P. and Anderson, D. R. (2002). *Model Selection and Multimodel Inference: A Practical Information-Theoretic Approach*. Springer Science & Business Media, Ney York, NY, USA.

Fieberg, J., Signer, J., Smith, B., and Avgar, T. (2021). A 'How to' Guide for Interpreting Parameters in Habitat-Selection Analyses. *Journal of Animal Ecology*, 90(5):1027–1043.

Fortin, D., Fortin, M.-E., Beyer, H. L., Duchesne, T., Courant, S., and Dancose, K. (2009). Group-Size-Mediated Habitat Selection and Group Fusion–Fission Dynamics of Bison under Predation Risk. *Ecology*, 90(9):2480–2490.

Hofmann, D. D., Behr, D. M., McNutt, J. W., Ozgul, A., and Cozzi, G. (2021). Bound within boundaries: Do protected areas cover movement corridors of their most mobile, protected species? *Journal of Applied Ecology*, 58(6):1133–1144. Publisher: Wiley Online Library.

Pomilia, M. A., McNutt, J. W., and Jordan, N. R. (2015). Ecological Predictors of African Wild Dog Ranging Patterns in Northern Botswana. *Journal of Mammalogy*, 96(6):1214–1223.

Signer, J., Fieberg, J., and Avgar, T. (2019). Animal Movement Tools (amt): R Package for Managing Tracking Data and Conducting Habitat Selection Analyses. *Ecology and Evolution*, 9:880–890.
